# Nanomedicine to Overcome Multidrug Resistance Mechanisms in Colon and Pancreatic Cancer: Recent Progress

**DOI:** 10.3390/cancers13092058

**Published:** 2021-04-24

**Authors:** Raúl Ortíz, Francisco Quiñonero, Beatriz García-Pinel, Marco Fuel, Cristina Mesas, Laura Cabeza, Consolación Melguizo, Jose Prados

**Affiliations:** 1 Institute of Biopathology and Regenerative Medicine (IBIMER), Center of Biomedical Research (CIBM), University of Granada, 18100 Granada, Spain; roquesa@ugr.es (R.O.); fjquinonero@ugr.es (F.Q.); beatrizgarnel@ugr.es (B.G.-P.); marcofh@correo.ugr.es (M.F.); cristinam@correo.ugr.es (C.M.); lautea@ugr.es (L.C.); jcprados@ugr.es (J.P.); 2 Department of Anatomy and Embriology, Faculty of Medicine, University of Granada, 18071 Granada, Spain; 3 Instituto Biosanitario de Granada (ibs.GRANADA), 18014 Granada, Spain

**Keywords:** drug resistance, colon cancer, pancreatic cancer, nanomedicine, cancer stem cells, PARP, miRNA, tumor microenvironment

## Abstract

**Simple Summary:**

The cellular mechanisms of drug resistance prevent the correct efficacy of the therapies used in various types of cancer and nanotechnology has been postulated as a possible alternative to avoid them. This review focuses on describing the different mechanisms of drug resistance and dis-covering which nanotechnology-based therapies have been used in recent years to evade them in colon (CRC) and pancreatic cancer (PAC). Here we summarize the use of different types of nanotechnology (mainly nanoparticles) that have shown efficacy in vitro and in vivo in preclinical phases, allowing future in-depth research in CRC and PAC and its translation to future clinical trials.

**Abstract:**

The development of drug resistance is one of the main causes of cancer treatment failure. This phenomenon occurs very frequently in different types of cancer, including colon and pancreatic cancers. However, the underlying molecular mechanisms are not fully understood. In recent years, nanomedicine has improved the delivery and efficacy of drugs, and has decreased their side effects. In addition, it has allowed to design drugs capable of avoiding certain resistance mechanisms of tumors. In this article, we review the main resistance mechanisms in colon and pancreatic cancers, along with the most relevant strategies offered by nanodrugs to overcome this obstacle. These strategies include the inhibition of efflux pumps, the use of specific targets, the development of nanomedicines affecting the environment of cancer-specific tissues, the modulation of DNA repair mechanisms or RNA (miRNA), and specific approaches to damage cancer stem cells, among others. This review aims to illustrate how advanced nanoformulations, including polymeric conjugates, micelles, dendrimers, liposomes, metallic and carbon-based nanoparticles, are allowing to overcome one of the main limitations in the treatment of colon and pancreatic cancers. The future development of nanomedicine opens new horizons for cancer treatment.

## 1. Introduction

According to the latest epidemiological data, colorectal cancer (CRC) and pancreatic cancer (pancreatic adenocarcinoma or PAC) rank third and eleventh in cancer incidence worldwide, respectively. In terms of cancer mortality, they are the second and seventh leading causes, respectively. Despite its low incidence, PAC has the highest mortality rate of all cancers, with a 5-year survival rate of only 9% [1,2]. On the other hand, CRC has a better prognosis in the initial stages of the disease, but in stage IV (i.e., metastatic) its mortality is also very high, with 5-year survival rates of only 13% [3]. In both PAC and stage IV CRC, current therapies only result in a slight increase in survival, mainly due to the phenomenon of drug-resistance. 

Drug-resistance can be classified into innate or acquired (generated after treatment) [4]. Innate resistance usually results from pre-existing mutations in genes involved in cell growth or apoptosis [5]. For instance, the *p53* mutation abolishes the function of this protein, facilitating resistance to standard medications used in colon and pancreatic tumors, such as gemcitabine (GEM) (antimetabolites), doxorubicin (DOX) (anthracyclines), or cetuximab (EGFR-inhibitor) [6]. Genes involved in DNA repair systems (e.g., MMR) may be altered in these tumors [7]. These systems are responsible for failure to standard neoadjuvant chemotherapy with 5-fluorouracil (5-FU)/oxaliplatin (OXA) and 5-FU-based adjuvant chemotherapy [8]. Another source of innate resistance is the activation of cellular pathways involved in the elimination of toxic elements or in damage prevention. These pathways can be exploited by tumor cells to protect themselves from anti-tumor drugs [5]. Examples of these are the transporter pumps, such as the ATP binding cassette (ABC) family members, including MDR1 or P-glycoprotein-P-GP- (ABCB1), Multidrug Resistance-Associated Protein 1 (MRP1) and Multidrug Resistance-Associated Protein 3 (MRP3) or Breast Cancer Resistance Protein (BCRP), which promote drug transport outside the cell in tumor pathology [9]. These existing pathways also include DNA repair enzymes such as poly(ADP-ribose) polymerase-1 (PARP-1), which allows cells to survive the DNA damage caused by both intrinsic and extrinsic factors, thus promoting drug resistance [10]. PARP-1 inhibition has been linked to the better response to therapy in various cancers, including PAC [11]. Finally, another source of innate resistance lies in the heterogeneity of the tumor, which may contain pre-existing cell subpopulations insensitive to treatment, including cancer stem cells (CSCs). Numerous trials have demonstrated the importance of CSCs in tumor resistance, since these cells are selected after chemo and radiotherapy [12], and may be resistant even to novel therapies, such as immunotherapy. 

The resistance developed after antitumor treatment is known as acquired resistance and causes a gradual reduction of the anticancer efficacy of drugs. This resistance may result from the development of new mutations (which may affect new proto-oncogenes or modify treatment targets) or from alterations in the tumor microenvironment (TME) during treatment, leading to decreased antitumor efficacy of drugs. An example of modification of the treatment targets are mutations of the EGFR ectodomain, which have been shown to generate secondary resistance to anti-EGFR monoclonal antibodies (mAbs) (i.e., rituximab) in CRC [13,14]. Regarding TME-related acquired resistance, the TME promotes the survival and migration of cancer cells, conferring resistance to chemo, radio and immunotherapy treatments [15]. In sum, resistance phenomena involve complex and diverse resistance mechanisms that can occur simultaneously during tumor development and treatment. Therefore, it is essential to search for new therapeutic alternatives to overcome these mechanisms.

Nanomedicine is based on the use of nanomaterials, taking advantage of their different physicochemical properties to develop innovative applications in the field of medicine. The use of these materials has allowed significant improvements in antitumor treatment through the generation of nanoparticles (NPs) capable of transporting and releasing the drug in tumor cells more efficiently. In addition, NPs can be conjugated with different ligands on their surface to specifically damage tumor cells and reduce drug toxicity in healthy tissues [16]. Remarkable advantages of these molecules include their ability to passively accumulate in tumors via enhanced permeability and retention (EPR) effect, which results from increased disorganization of the vasculature and impaired lymphatic drainage of tumors, and their stability in blood due to improvements such as pegylation [17]. In this review, we will examine the different drug-resistance mechanisms of tumours and how the use of nanomedicine and, specifically, NPs can improve the treatment of CRC and PAC. 

## 2. Increased Efflux of Drugs

### 2.1. Efflux Pump P-Glycoprotein and Drug Resistance

One of the most relevant resistance mechanisms of cancer cells is P-GP, a membrane protein which pumps out the drugs from the inside of cells to the extracellular space, reducing their therapeutic effect [18]. This protein, also known as ATP-binding cassette subfamily B member 1 (ABCB1) or ABC transporters, comprises a superfamily of efflux pump proteins with different members that may be involved in the MDR phenotype of tumor cells. In addition to P-GP, other members from this superfamily play an important role in the MRD phenotype in CRC and PAC, such as MDR-associated proteins (MRPs/ABCCs) and breast cancer resistance protein (BCRP/ABCG2) [9,19] and MDR1 [20,21]. P-GP is a 170 kD protein normally present in a variety of cells of the digestive system (e.g., common bile duct of the liver and pancreatic ducts), being highly expressed in the apical surface of epithelial cells [20]. However, the overexpression of P-GP in cancer cells is usually associated with a low therapeutic efficacy [22,23,24,25,26,27] especially in CRC. In this context, many nanoformulations have been designed to overcome the P-GP-mediated MDR phenotype in CRC and PAC (Table 1). 

**Figure 1:**
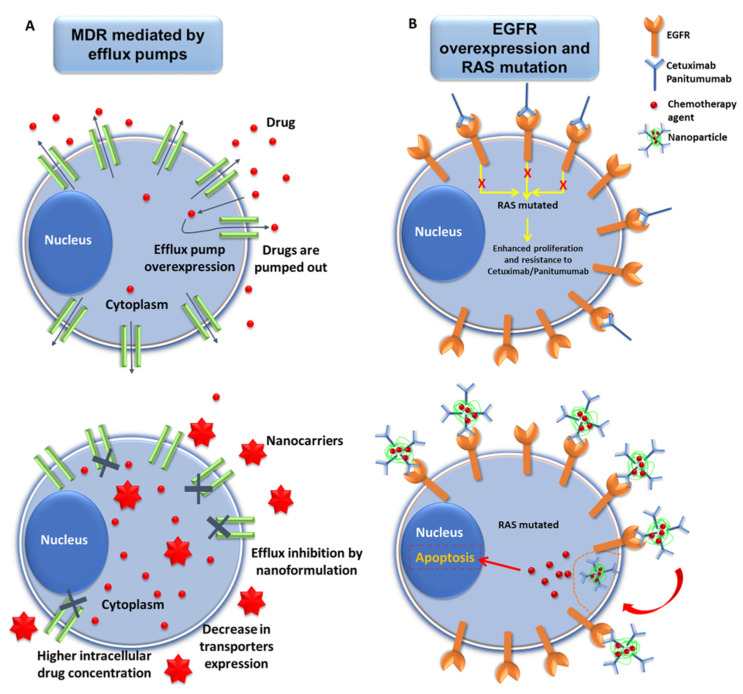
Representative scheme of nanomedicine strategies against drug resistance in pancreatic cancer. (**A**) Interactions of nanoformulations with MDR mechanism in pancreatic cancer (PAC) cells. Drugs carried by NPs may overcome MDR mechanisms of PAC cells mediated by efflux pumps via the inhibition of drug efflux, decrease in the expression of the transporter proteins, and increased accumulation of drug inside the tumor cells. (**B**) Exploiting the overexpression of EGFR to overcome resistance mediated by RAS mutations in PAC cells. PAC cells overexpress EGFR, which can be targeted by NPs conjugated with mAbs (Cetuximab/Panitumumab) (see below). This enables increased internalization of the chemotherapy drugs, thus leading to cellular apoptosis, even in RAS-mutated cancer cells.

Despite the fact many P-GP inhibitors have been developed (e.g., verapamil, tariquidar, 11C-laniquidar, natural alkaloids or herbal natural products) [55,56,57,58,59], most of them showed significant limitations (systemic toxicity, insolubility, short blood half-life or rapid metabolization) [21,58,60,61]. The use of nanoformulations could overcome these drawbacks (Figure 1A). Recently, bufalin (BU), an antitumor drug that blocks P-GP-mediated resistance in CRC [62], was associated with vitamin E succinate-grafted-chitosan oligosaccharide with RGD peptide (arginine-glycine-aspartic acid) and TPGS. Compared to free BU, this combination showed improved antitumor activity (43% tumor volume reduction), pharmacokinetic profile and toxicity in resistant-CRC (LoVo/ADR cells)-bearing nude mice [31]. Moreover, the nanoformulation alone (i.e., without P-GP inhibitors) can overcome the P-GP-mediated MDR. For instance, treatment of PANC-1, a PAC cell line that overexpresses P-GP with DOX-loaded liposomes modified with phage fusion proteins for specific targeting, showed similar drug accumulation in presence and absence of verapamil [63,64]. Accordingly, this nanoformulation avoided P-GP-mediated expulsion and increased cytotoxicity of DOX (IC_50_ 10-fold lower) [47]. Pan, et al. [65] overcame MDR mediated by P-GP in HCT8/ADR resistant cells using a large nanoformulation consisting of DOX-loaded poly(aspartic acid) NPs functionalized with TAT peptide and PEG, which is not a substrate of the efflux pump. In other cases, multifunctional NPs that combine several strategies at the same time to overcome the MDR phenotype have been used. For example, DOX-loaded gold nanorods coated with mesoporous silica (mSiO_2_) used in CRC photothermal therapy (PTT) and chemotherapy have been improved by using pH-responsive polyhistidine (PHIS) and D-α-tocopherol PEG 1000 succinate (TPGS) to induce endocytic pathway escape and to inhibit P-GP, respectively [28]. These nanorods showed excellent results in male athymic nude mice bearing SW620/Ad300 cells, achieving a significant reduction in tumor volume (~3-fold) with low systemic toxicity. Interestingly, resistant CRC has also been treated with local PTT using gold nanorods coated with PEG and paclitaxel (PTX) nanocrystals coated with TPGS (all of them combined into a hydrogel). Promising results were obtained in the SW620/Ad300 cell line in vitro (~178-fold decrease in the IC50 of PTX) and in male athymic nude mice bearing SW620/Ad300 tumors [30]. The use of phosphatidylserine (PS) lipid nanovesicles with encapsulated PTX showed a synergistic effect against the chemoresistant HCT15 cell line overexpressing the MDR1 gene product P-GP, both in vivo an in vitro. These NPs induced cell cycle arrest at G2/M phase, downregulated *ki-67*, *Bcl-2*, and *CD34*, upregulated caspase 3, reduced the systemic toxic effects of PTX and no inflammatory response was reported [66].

### 2.2. Other Efflux Pumps

Breast cancer resistance protein (BCRP/ABCG2), also known as mitoxantrone resistance protein (MXR) or placenta ABC protein (ABC-P), is a small member of the ABC superfamily (~75 KDa) with a more limited number of substrates (e.g., amptothecin [CPT], tyrosine kinase inhibitors and methotrexate) and inhibitors (e.g, imatinib or poloxamines) compared to P-GP [67]. Although BRCP/ABCG2 is usually highly expressed in hematological cancers, it has also been found to be overexpressed in CRC and PAC [68,69]. In fact, 72% of pancreatic cell lines from the Cancer Cell Line Encyclopedia (CCLE) overexpressed this protein [69]. Nanoformulations such as irinotecan-loaded liposomes with benzoporphyrin derivative for photodynamic therapy (PDT) were able to overcome ABCG2-mediated resistance, decreasing tumor volume from 70% to 25% in mice bearing pancreatic tumors [41]. PDT has also been used with polymeric NPs with PEG and polyethylenimine (PEI) in pancreatic cells with variable expression levels of ABCG2 (+AsPC-1 and -MIA PaCa-2 and induced +MIA PaCa-2), increasing the intracellular concentrations of the photosensitizer through reduction of its efflux by ABCG2 [37]. On the other hand, pegylated poly-lactic-co-glycolic acid (PLGA) NPs with verapamil and SN38 (active form of CPT) showed no significant differences in drug cytotoxicity in HT29 CRC cells, but a significant decrease in ABCG2 mRNA expression (2.277-fold) in comparison with the free drug (4.793-fold) was observed [35]. In addition, microbubbles containing porphyrin/CPT-floxuridine for chemotherapy, PDT and imaging, generated NPs suitable for tumor therapy after exposure to ultrasound. This treatment significantly reduced the expression of ABCG2 in HT29 cells by increasing the concentration of CPT, inducing an in vivo growth inhibition (90%) of the tumor without recurrence [70].

On the other hand, the MRP1/ABCC1 and MRP3/ABCC3 proteins, which have a similar molecular weight (~190 KDa) and structure, contain a different n-terminal region in relation to P-GP [71,72,73]. Drug substrates of MRP1 and P-GP are similar except for taxanes, which are only P-GP substrates [73]. The spectrum of molecules transported by MRP3 is limited [74] In fact, MRP1 is the protein mostly involved in the therapeutic failure resulting from MDR [75]. Some nanoformulations have been designed to specifically overcome efflux pumps, including MRP. Nitric oxide-releasing DOX (NitDOX) was loaded in liposomes to treat HT29 cells resistant to DOX mediated by MRP1 and P-GP. Nitration of both proteins significantly reduced their activity [48]. In addition, pegylated liposomes loaded with epirubicin and antisense oligonucleotides (ASOs) against MDR1, MRP1 and MRP2 increased the antitumor activity of the drug in mice bearing CRC (CT26 cells), while administration of ASOs alone did not show significant differences between resistant and non-resistant tumors [51]. 

## 3. Alteration of Drug Target

Targeted therapy uses drugs that damage tumor cells and specific targets such as genes, proteins, or the environment of cancer-specific tissues, all of which contribute to cancer growth and survival. However, cancer cells can develop resistance by altering these pharmacological targets, either through genetic mutations or via changes in epigenetic gene expression [76].

### 3.1. Epidermal Growth Factor Receptor (EGFR) Pathway

The epidermal growth factor receptor (EGFR), also known as ErbB1 or HER1, is a transmembrane glycoprotein of the tyrosine kinase family with the epidermal growth factor as a ligand. Although present in the majority of cells, this protein is a target for cancer therapy because of its overexpression, amplification and mutation in a wide variety of solid tumors [5,77]. EGFR-targeting agents clinically used in malignancies include mAbs against the extracellular domain of the receptor, and small molecules (tyrosine kinase inhibitors). However, EGFR mutations, such as EGFRvIII, which is characterized by the loss of part of the extracellular ligand binding domain due to deletion of exon 2-7, may generate resistance to treatment [78,79]. 

Although CRC and PAC rarely show EGFR mutations, both cancers overexpress this receptor and have been treated with cetuximab or panitumumab and erlotinib, respectively [80]. However, overexpression of EGFR does not imply dependence of cancer cells on this receptor for oncogenic signaling. In fact, KRAS-mutated cancer cells do not require EGFR activation. Therefore, this mutation predicts resistance to EGFR-targeted therapy [81,82,83]. As described by Van Emburgh, et al. [84], 60% of patients with resistance to cetuximab or panitumumab showed the KRAS mutation. In this context, nanoformulations loading anticancer agents and mAbs have been developed, with the nanoconjugation design being a critical step for therapy (Figure 1B). For instance, Khan, et al. [85] used gold NPs associated with cetuximab against PAC cells. More recently, McDaid, et al. [86] used polymeric NPs loaded with camptothecin and conjugated with cetuximab against cetuximab-resistant PAC cells with mutated KRAS. This treatment produced a greater reduction in tumor growth than each of the drugs alone in mice, demonstrating the potential applicability of this therapeutic strategy. Functionalization of NPs with anti-EGFR antibodies was another solution to transport new non-water soluble anti-cancer compounds. Parvifloron D, isolated from a plant extract, is a hydrophobic drug with significant antitumor activity. Its conjugation with albumin NPs functionalized with anti-EGFR antibodies (erlotinib and cetuximab) made possible the treatment of PAC cells [87]. In CRC, gold NPs loaded with 5-FU and functionalized with cetuximab induced more apoptosis than the free drug in HCT-116 and HT-29 CRC cells [88]. These results were supported by those of Leve, et al. [89]) in HCT-116 CRC cells using similar nanoconjugates. 

Finally, some peptides such as GE11 have been used to target the EGFR. GE11 conjugated with NPs loaded with GEM and olaparib was used against PAC with mutated BRCA2, obtaining favorable results both in vitro and in vivo [90]. Similarly, a significant increase in cytotoxicity was obtained using PLGA gelatin NPs functionalized with an EGFR-targeting peptide and loaded with GEM in an orthotopic PAC animal model [91]. As is the case with PAC, polyamino acid NPs with GE11 peptide have been used to transport evodiamine, an indolequinone alkaloid which is highly effective against CRC but with low solubility. This nanoformulation induced a significant increase in drug cytotoxicity in vivo, along with a drastic reduction of metastases and GFR, VEGF and MMP protein expression [92].

### 3.2. Vascular Endothelial Growth Factor Pathway

The vascular endothelial growth factor (VEGF), a promoter of tumor angiogenesis and, consequently, and inductor of metastasis and proliferation, has been used as a target to reduce tumor growth. The use of NPs may improve the antiangiogenic effect of treatments. For instance, Leng, et al. [93] obtained excellent in vitro and in vivo results in CRC through the functionalization of calcium phosphate NPs loaded with 5-fluorocystone and with genetic sequences that inhibit the expression of VEGF (Lovo cell line). Lee, et al. [94] exposed a system of theragnostic nanoplatforms functionalized with small interfering RNA (siRNA) which located and blocked the cells that produce this factor in large quantities. The incorporation of campothecin (SN-38) to these NPs allowed a selective effect in CRC cells.

## 4. Enhanced DNA Damage Repair

The development of small mutations in essential genes involved in cellular regulation, such as tumor suppressor genes (e.g., *p53*) and chromosomal instability are among the primary reasons underlying the formation of new tumors [95]. Genomic stability is maintained by highly efficient replication and damage repair mechanisms [96]. However, these mechanisms may be related to drug resistance. In fact, the inhibition of DNA repair pathways has also been advocated to increase the efficacy of treatment in various types of cancer. In this context, the PARP family and MMR, involved in the repair of DNA damage, have been explored to avoid MDR using nanotechnology.

### 4.1. PARP Related Mechanisms

One of the first events produced after DNA damage is the recruitment of the PARP1 enzyme, a protein capable of binding poly-ADP ribose to different proteins, including itself. This binding allows the recruitment and stabilization of protein complexes involved in DNA damage repair through different pathways, namely single-strand break repair (SSBR), nucleotide excision repair (NER) and base excision repair (BER). Furthermore, PARP is involved in the repair of double-strand breaks in DNA via homologous and non-homologous end joining (NHEJ), and also participates in the modulation of the structure of chromatin, which implies a great influence on the regulation of important cellular processes [97]. The relevance of PARP1 in all these processes has prompted the development of several inhibitors to avoid efficient repair of DNA damage which, together with mutations produced in other repair pathways, can prevent resistance to pharmacological treatment.

**Figure 2:**
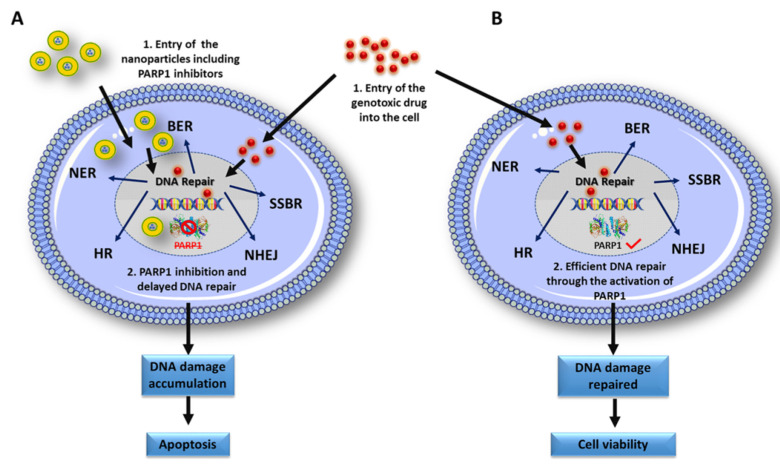
Nanoformulations to fight against drug resistance induced by PARP1. PARP1 modulates DNA damage repair by single-chain (SSBR, BER and NER) and double-chain (HR and NHEJ) repair pathways. PARP inhibitors are used to avoid drug resistance by limiting PARP1 functions in the different DNA repair pathways in which this protein is involved and therefore nanoformulations containing PARP inhibitors have been designed for different types of cancer. (**A**) The simultaneous entry of nanoparticles containing PARP1 inhibitors together with the genotoxic drug causes the inactivation of PARP1, necessary for the efficient repair of DNA damage through all the routes in which it is involved. This causes that the damages produced by the genotoxic agent in the DNA are not repairable, inducing cellular apoptosis.; (**B**) Entry of the genotoxic agent into the cell produces easily repairable DNA damage through DNA repair pathways where PARP1 acts as an important effector, leading to cell survival. Abbreviations: homologous recombination (HR).

The use of PARP1 inhibitors associated with NPs has been assayed in some cancers such as hepatocellular carcinoma (arsenite and DOX) [98], prostate cancer (nano-olaparib) [99], ovarian cancer (NPs loaded with olaparib and cisplatin) [100] and, especially, breast cancer. Regarding the last group, the use of nanoliposomes loaded with a PARP inhibitor (talazoparib) in BRCA-negative breast cancer [101] and the use of talazoparib-loaded solid lipid NPs (LPNs) in triple negative breast cancer [102] demonstrated enhanced therapeutic efficacy (Figure 2). Moreover, PAC has also been associated with BRCA1/2 mutations and there are ongoing early-phase studies using PARP1 inhibitors in this cancer. In fact, talazoparib was studied in patients with advanced PAC [103]. Recently, NPs loaded with GEM and Olaparib were used to specifically target BRCA2-negative cells that overexpress EGFR. Encapsulation and functionalization of NPs allowed to increase the half-life in blood of drugs and their accumulation within the tumor tissue [90]. The use of PARP inhibitors with NPs in CRC has not been exploited. In fact, early-phase clinical studies using PARP1 inhibitors (specifically Olaparib) in metastatic CRC showed no significant efficacy. Authors leave open the possibility of using PARP inhibitors together with chemo- or radiotherapy with the aim to improve the effectiveness of treatment [104]. It is still necessary to determine the influence of PARP on drug resistance in CRC, in addition to the development of new NPs that allow an efficient transport of new inhibitors. Therefore, further studies are required in this type of cancer.

### 4.2. MMR Based Mechanisms

The MMR system is responsible for repairing errors generated by DNA polymerases during the process of DNA replication and recombination. Proteins such as MLH1, MHS2, MHS6 and PMS2 interact as heterodimers in this process in order to recognize mismatches and insertion-deletion errors, eliminating the erroneous DNA fragment and resynthesizing the correct strand [105]. The MMR system repairs damage caused by both endogenous and exogenous factors (ionizing and UV radiation, chemical agents, toxins, chemo/radiotherapy, or environmental stress) [106]. It has been demonstrated that mutations of certain genes involved in these pathways (e.g., BRCA1, BRCA2, PALB2 and MSH2) lead to lower DNA repair in the tumor, increasing the efficiency of drugs and the survival of patients [107]. 

The MMR system has been associated with CRC. For instance, epigenetic changes of germline MMR genes (MSH2, MLH1, MSH6 and PSM2) have been correlated with Lynch syndrome (1/35 cases of CRC) [108]. In addition, CRC cell lines with MLH1 mutations showed a higher sensitivity to treatment with irinotecan, increasing DNA damage. The addition of PARP inhibitors increases the damage produced, leading to extensive apoptosis in the population of tumor cells [109]. The use of iron particles reduced the expression of two MMR genes (PMS1 and MSH2) in lung cancer, inducing defective DNA repair. However, the employment of nanotechnology to inhibit the MMR pathways is anecdotal in tumor pathology, including CRC [110]. Although the mechanisms of DNA damage repair based on MMR and other pathways have been extensively studied, further research on new inhibitors is needed to increase the sensitivity of chemo/radiotherapy in cancer, in addition to the development of NPs that allow the correct function and efficient distribution of these inhibitors.

## 5. Pro- and Anti-Apoptotic Genes: Evasion and Overexpression 

Cancer cells develop strategies to decrease the activity of pro-apoptotic proteins or increase the activity of anti-apoptotic proteins [111], avoiding programmed cell death and generating resistance. NPs have been used with the aim to modulate the Bcl-2 protein family, which includes both anti-apoptotic (e.g., *Bcl-2, Bcl-XL, Mcl-1*) and pro-apoptotic members (e.g., *BAX, Bak*), among others [112]. Metal NPs such as silver NPs (AgNPs) have been reported to cause downregulation of anti-apoptotic genes (*Bcl-2* and *Bcl-XL*), and upregulation of pro-apoptotic genes (*Bax, Bad* and *Bak*) in HCT116 and CaCo2 CRC cells, and in HT-29 lung cancer cells [113,114,115,116,117]. In addition, these NPs led to an increased expression of the *p53* and p21 genes and to higher levels of caspase 3, inducing *p53*-mediated apoptosis [114,117]. A similar effect was observed in PANC-1 cells with the use of AgNPs, which decreased the expression of Bcl-2 while increased that of Bax, *p53* and autophagy proteins (RP-1, RIP-3, MLKL and LC3-II) [118]. In addition, a nanoemulsion system incorporating lycopene (LP) and gold NPs (AN) reduced proliferation in HT-29 cells mediated by a significant decrease in the expression of procaspases 8, 3, 9, PARP-1 and Bcl-2, whereas the Bax expression increased [119]. Magnetic gold NPs loaded with siRNA have been used to decrease the expression of Bag-1, another anti-apoptotic gene highly expressed in colon cancer cells (LoVo cells) [120]. Furthermore, copper oxide NPs (CuO-NPs) inhibited the proliferation of HT-29 and SW620 cancer cells by decreasing the expression of Bcl-2 and Bcl-xL [121]. Finally, copper cysteamine (Cu-cy) NPs activated by X-rays have been used against SW620 CRC cells, producing apoptosis by autophagy due to the increased expression of *Bax*, *LC3B-II* and *p62*, and the decreased expression of *Bcl-2* [122]. 

On the other hand, certain toxic anticancer agents that influence the expression of pro-apoptotic or anti-apoptotic proteins were conjugated with NPs to tackle this resistance mechanism. Accordingly, arsenic trioxide encapsulated in an ethylene glycol/poly D,L-lactide copolymer and conjugated with anti-CD44v6 led to decrease *Bcl2* and increased caspase 3 levels in PANC-1 PAC cells [123]. In Caco2 and HeLa cancer cells, orthorhombic tungsten oxide NPs decreased cell viability by 65% and 73%, respectively, by reducing the expression of *Bcl-2* and *MMP7* [124]. Motawi, et al. [125] reported that cromolyn chitosan NPs led to a decreased expression of Bcl-2, NF-kβ and an increased expression of Bax in dimethylhydrazine-induced CRC in rats. Finally, some natural products from marine or plant resources associated with NPs can also affect the expression of apoptotic proteins. Fucoxanthin (FUCO), a marine carotenoid, in combination with nanogels (NG) of chitosan (CS) and glycolipids (GL), reduced the expression of Bcl-2 and increased the activity of *Bax*, ROS and caspase 3 in Caco-2 cells [126]. Gambogic acid, another natural compound extracted from gamboge, conjugated with magnetic NPs (MN-Fe304), induced the modulation of *Bcl-2, iBax*, and caspases 9 and 3 in Capan-1 PAC [127]. In addition, a dendrosomal curcumin nanoformulation increased the expression of *BAX, Noxa* and *p21* and decreased that of *Bcl-2* in *p53*-mutant SW480 colon cancer cells. Moreover, curcumin-loaded magnetic NP formulations were tested in HPAF-II and PANC-1 cells, decreasing cell proliferation, both in in vitro and in vivo models. Also, these NPs reduced the tumor volume and downregulated *Bcl-XL, Mcl-1*, *PCNA*, *MUC1* and collagen I, and upregulated the expression of β-catenin [128].

## 6. RNAs in Drug Resistance

In recent years, the importance of the non-coding portion of the genome in the development of diseases has been recognized. Non-coding RNA (ncRNA) comprises different members such as piRNAs, snoRNAs or lincRNAs. However, two ncRNAs, namely lncRNA and miRNA, are known to have the highest incidence in pathologies. Dysregulation of their expression levels can cause drug resistance in various types of cancer [129]. LncRNAs play an important role in regulating mRNA stability, in RNA splicing and in genetic regulation of miRNAs. In PAC, various lncRNAs have been linked to epithelial mesenchymal transition (EMT) processes, regulation of CSCs, cellular hypoxia, modulation of epigenetic modifications, chemo-resistance, and regulation of the tumor microenvironment [130,131]. In CRC, deregulation of the XIST lncRNA decreased DOX resistance through its influence on the regulation of miR-124 and SGK1 protein expression [131]. Other lncRNAs such as UCA1 and CACS15 confer resistance to 5-FU and OXA, respectively, through the regulation of relevant miRNAs and genes involved in damage resistance pathways (miR-204-5p, miR-145 and the ABCC1 protein) [132,133]. On the other hand, miRNAs, the most studied ncRNAs, are involved in the regulation of approximately 30% of human genes (cell cycle regulation, proliferation, and stress tolerance), including those related to drug resistance in different cancers such as breast, ovarian, colorectal, pancreatic and gastric, among others [134]. Specifically, the deregulation of 16 miRNAs has been described in CRC, and the analysis of patients with MDR tumors determined that some of these miRNAs were overexpressed in exosomes obtained from the blood serum of the said patients. Nanotechnology and miRNAs may be the basis to develop new therapeutic options to improve cancer prognosis [135] (Table 2). 

### 6.1. miRNAs in Colorectal Cancer

miR-375-3p targets the thymidylate synthase (TYMS) enzyme, which regulates drug resistance to 5-FU. The expression of TYMS was found to be increased in HCT116 cells and in the 5-FU-resistant cell line HCT-15/Fu. miR-375-3p was downregulated in the colon cancer cell lines SW480, HCT116, HT29 and Caco2 compared to the non-tumor colon cell line NCM460. Xu, et al. [136] demonstrated that miR-375-3p associated with 5-FU and loaded in calcium carbonate and lipid-coated NPs improved chemo-sensitivity to 5-FU and inhibited cell proliferation, both in vitro and in vivo. A similar effect was described using miR-200 associated with Irinotecan-loaded liposomes coated with PEG peptides, which reduced the growth of HCT116 colon cancer cells. This effect was also observed in tumor-bearing mice through the suppression of EMT, MDR, B-catenin and apoptosis signaling pathways [137]. 

Other miRNAs associated with a nanosystem were assayed in colon cancer treatment, including miR-204-5p, miR-145, miR-139, miR-21 and miR-155. The miR-204-5p, an important tumor suppressor factor, is downregulated in colon cancer. This miRNA was incorporated in pegylated polymer NPs, showing an antiproliferative effect in HCT-116 colon and HT-29 lung cancer cells in a tumor xenograft model [139]. miR-145, also downregulated in colon cancer cells, and PLGA/PEI/HA nanocomplexes were used to increase the expression of miRNA-145 in the tumor tissue of a xenograft model from HCT-166 colon cancer cells. This treatment induced cell cycle arrest in the G0/G1 phase, decreased tumor proliferation and migration, and increase apoptosis [140]. As mentioned above, EGFR is known to be overexpressed in colon cancer. miR-139 was combined with afatinib, an oral tyrosine kinase and EGFR inhibitor, into lipid polymeric NPs conjugated with a targeting ligand and a pH-sensitive penetrating peptide (Afa/LPN-HR). The results showed that these NPs induced apoptosis, inhibited cell migration and decreased drug resistance to Afatinib in Caco-2 cells by modulating the *EGFR, HER, Ras, Akt, Stat4, Mapk*, EMT and *Bcl* pathways [141]. Finally, some systems have been developed to inhibit oncogenic miRNAs. A nanosystem consisting of fluorescent nanodiamond and antisense RNA was used to eliminate the oncogenic microRNA-21 in CT-26 colon cancer cells. The effect was the activation of the *PDCD4* and *Timp3* tumor suppressor genes, resulting in a decrease in cell invasion, migration and induction of apoptosis [142]. In addition, a nanoplatform consisting of anti-miR-155 loaded in mesoporous silica NPs (MSNs-anti-miR-155-PDA-Apt) was used to reduce the expression of miR-155 in SW480 cells and in in vivo experiments. This nanoplatform exhibited an antiproliferative effect due to the active targeting of the AS1411 aptamer and passive targeting of the EPR effect. In addition, they re-sensitized 5-FU-resistant tumors by reducing p-GP [143].

### 6.2. miRNAs in Pancreatic Cancer

Nanoformulations using miRNAs (e.g., miR-145, miR-21, miR-216b, miR-210, miR-634, miR-211 or miR-210) to overcome drug resistance have also been tested in PAC. HPAF-II and AsPc-1 PAC cells showed downregulation of the miR-145 tumor suppressor miRNA. In addition, a magnetic NP formulation (miR-145-MNOF) was able to restore and increase miRNA expression, reducing the levels of *MUC13, HER2* and *pAKT*, and inhibiting cell proliferation, migration and invasion [145]. Li, et al. [152] used miR-21 antisense oligonucleotides (ASO-miR-21) and GEM associated with PEG-polyethylenimine-magnetic iron oxide NPs and an anti-CD44v6 single-chain variable fragment against PANC-1 and MIA PaCa-2 PAC cells. This system decreased the expression of miR-21 and increased the expression of the *PDCD4* and *PTEN* tumor-suppressor genes. In the same two cell lines, single-stranded (SS) miR-216b included into palmityl-oleyl phosphatidylcholine (POPC) liposomes conjugated with two palmityl chains and a cell penetrating peptide (TAT) was able to restore the expression of miR-216b, which was downregulated. Moreover, this system suppressed the oncogenic *KRAS* and inhibited colony formation in both PANC-1 and MIA PaCa-2 cell lines [146]. *KRAS* is a proto-oncogene frequently mutated in pancreatic ductal adenocarcinoma (PDAC). The possibility of blocking oncogenic pathways involving *KRAS*-induced *ERK/AKT* signaling is a promising therapeutic strategy. Mokhlis, et al. [149] overexpressed miR-873 in Capan-2, Mia Paca-2, PANC-1 and BxPc-3 pancreatic cell lines and in an orthotopic xenograft mouse model to induce apoptosis. In addition, dual targeting of miR-21 (anti-miR-21) and *KRAS* (siKRAS or mimic-217) packaged into the tumor-penetrating NPs decreased tumor growth in in vivo models generated from PANC-1 and D8-175 PDAC cell lines [153]. The administration of si-KRAS and anti-miR-210 loaded in cholesterol NPs in an in vivo model modulated the TME, led to a delay of tumor growth and inhibition of metastasis, and prolonged survival [154]. microenvironment is especially relevant in PADC due to its lack of vascularization and the presence of a dense stroma. On the other hand, the enforced expression of some miRs such as miR-634 can induce apoptosis in cancer cells by modulating the expression of genes associated with anti-apoptotic signaling, mitochondrial homeostasis, cytoprotective processes and autophagy. In this context, LNPs harboring miR-634 showed therapeutic potential against a xenograft tumor from BxPC-3 PAC cells in mice [150]. 

Finally, peptides targeting plectin (PL-1), a novel biomarker for PAC, have shown high specificity for PDAC in in vivo experiments. A chimeric peptide (PL-1) associated with miR-211 in supramolecular NPs decreased the expression of USP99X and enhanced the effect of DOX (apoptosis and autophagy) on CFPAC-1, CAPAN-1, PAN-198 and PANC-1 PAC cells [147]. A similar system using miR-9 improved the anticancer effect of DOX by downregulating the expression of Eif5a2 in CFPAC-1, CAPAN-1, PAN-198 and PANC-1 cells and in a PAC patient-derived xenograft model [148]

## 7. Epigenetic Alterations 

Although many diagnosed tumors are caused by genetic factors, these do not explain all of the cases. Epigenetics studies have shown that the methylation of essential genes involved in an adequate regulation of cells (e.g., *p53*, *APC, RAS, PTEN* or *Wnt*) gives rise to genetic instability, which ends up causing the development of CRC [155]. Similarly, epigenetic dysregulations related to the *p16, PTEN* and *RAS* genes, among others, can be found in certain cases of aggressive PAC [156,157]. In this context, epigenetic modifications and the existence of non-coding regulatory RNAs in our genome may influence drug resistance, becoming a potential target to improve cancer treatment.

There is a wide variety of epigenetic modifications that can occur in the DNA, histones and in nucleosome remodelling, but the most studied are methylation and acetylation. DNA methylation can inactivate genes which are necessary for the maintenance of the basal state of tissues, causing deregulation of tumor suppressor genes and leading to the development of CRC and PAC [158]. Cell lines resistant to 5-FU showed epigenomic changes in the expression of genes influencing the process of resistance [159]. Other epigenetic modulations that induce downregulation of the PCAF acetyltransferase increased resistance to 5-FU in colon cancer by reducing drug-induced apoptosis [160]. In addition, the overexpression of the arginine methyltransferase 3 (PRMT3) protein, which allows the expression of the *ABCG2* gene, increased chemoresistance in PAC cell lines [161]. 

Since DNA methylation is one of the modifications most frequently involved in cancer (including CRC and PAC), several treatment strategies focused on this epigenetic process have been designed. Accordingly, Gd-metallofullerenol nanomaterials were able to inhibit the interaction between histone deacetylase I (HDAC1) and metastasis-associated protein (MTA1), suppressing cell invasion and metastasis in PAC [162]. Valproic acid, an HDAC2 inhibitor, was encapsulated in polysaccharide NPs, allowing its hemocompatibility and avoiding toxicity [163]. In addition, other HDAC inhibitors, such as vorinostat and quisinostat, were associated with PLGA-lecithin-PEG NPs to increase the radiosensitization of these tumors [164]. More recently, Kularatne, et al. [165]) developed polycaprolactone polymer NPs associated with 4-phenylbutyric acid, an HDAC inhibitor, to enhance DOX-based therapy in CRC, while Busi, et al. [166] developed keratin NPs loaded with 9-hydroxystearic acid, another HDAC1 inhibitor, which induced cell cycle arrest and cell death in CRC lines.

## 8. Drug Resistance and Tumor Microenvironment

**Figure 3:**
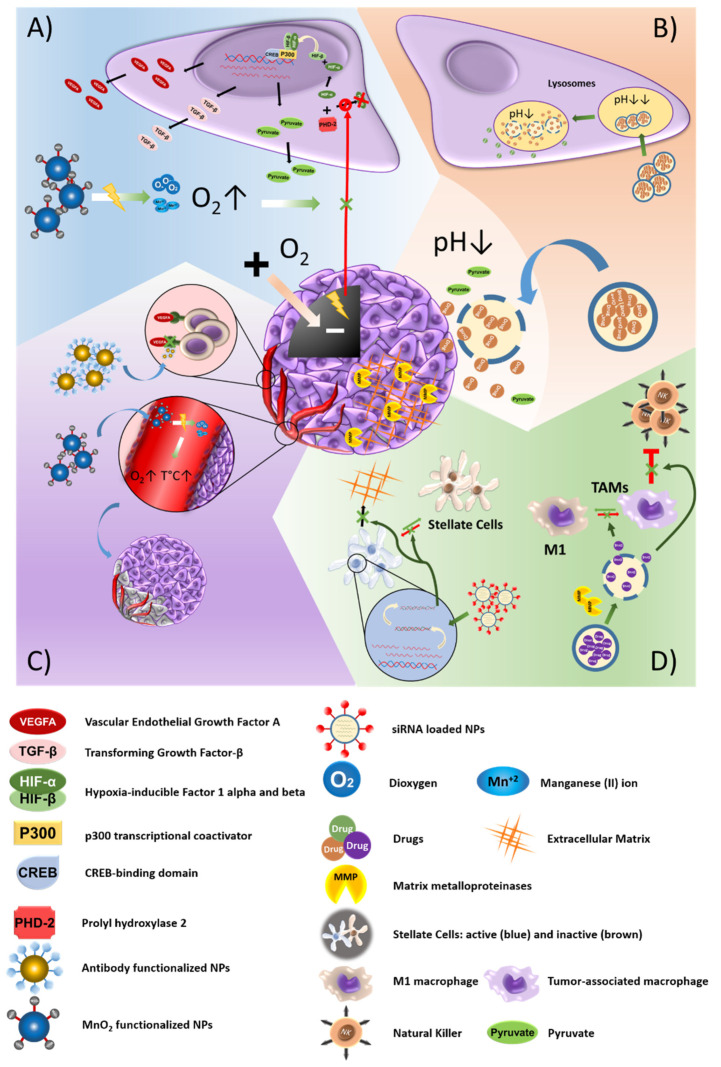
Nanoparticle-based strategies to exploit the characteristics of the tumor microenvironment (TME). (**A**) Systems that focus on the presence of hypoxic areas are based on the generation of oxygen, increasing its local levels and, therefore, eliminating the stress situation which results from hypoxia. Reversal of the hypoxic state blocks the expression of *HIF-1α* and, consequently, reduces the acidification of the extracellular medium and the expression of pro-angiogenic factors. (**B**) Medium acidification can be exploited by using pH-sensitive nanoplatforms with the aim of both achieving a controlled release of their load and buffering the medium to reestablish the pH. (**C**) Some strategies also focus on the active targeting of the neo-vasculature through the use of NPs that specifically recognize it by means of antibodies and the application of radiation. (**D**) In addition, some cellular components of the TME can be reprogrammed by using certain drugs, or inactivated by vehiculation of siRNA.

The TME is a highly complex and heterogeneous set made up not only of cancer cells, but also of immune system and epithelial cells, in addition to the substances which these secrete. The feedback between tumor cells and their environment allows reciprocal adaptations in order to overcome stressful situations and increase tumor survival [167]. Most tumors contain hypoxic areas in which the hypoxia-inducible factor 1 alpha (*HIF-1α*) is overexpressed. This factor is responsible for the transcription of numerous genes related to altered metabolism, angiogenesis, or metastatic progression [168,169]. HIF-1α can lead to an accumulation of pyruvate and nicotinamide adenine dinucleotide (NADH) and induction of the expression of lactate dehydrogenase A (LDHA), increasing the levels of lactate in the extracellular medium (ECM), with its subsequent acidification [168]. Hypoxia can also induce angiogenesis in the surrounding endothelial cells by releasing the *VEGF-A*, or promote myofibroblast differentiation through the expression of transforming growth factor-β (*TGF-β*) [169,170,171]. Thus, hypoxic areas favor the expression of drug resistance factors that lead to decreased effectiveness of treatments. Some strategies based on the use of NPs focus on hypoxic-related conditions, pH status or neovacularization to improve the action of drugs and avoid resistance (Figure 3). Javan, et al. [172] based their approach on the development of a hypoxia-sensitive system. The authors created a vector composed of shRNAs for *β-catenin* and *Bcl-2* driven by the promoter for carcinoembryonic antigen (CEA) and *VEGF*, loaded in PEI/Thiolated-chitosan NPs. In hypoxia conditions, this system inhibited the expression of *Bcl-2* and *β-catenin* by 51% and 56%, respectively, and increased the rates of apoptosis by 40% in HT29 CRC cells. In addition, hypoxia-sensitive systems have been assayed in CRC to increase the oxygen levels, reducing hypoxia and, consequently, drug resistance. In fact, Meng, et al. [173] functionalized human serum albumin (HSA)-PTX-NPs with MnO2, increasing the concentration of O2 and destabilizing HIF-1α (58.93% reduction) in mice bearing CT26 tumors. Subsequent radiation of the tumor achieved a synergistic effect due to increased tumor oxygenation, inhibiting tumor growth by 96.57%. Furthermore, gold nanocages functionalized with MnO2 and hyaluronic acid (HA) eliminated hypoxic areas and boosted O2 production after near-infrared (NIR) irradiation in CRC [174]. Aljabali, et al. [175] used piceatannol (resveratrol analog) associated with bovine serum albumin (BSA) to reduce the expression of *HIF-1α* and *p62* in CRC (Caco-2 and HT29 cells).

In PAC, iRGD-functionalized pegylated polymersomes associated with azobenzene improved the release of GEM in hypoxic conditions, both in vitro and in vivo [176]. In addition, Chen, et al. [177] aimed to overcome the limitations of hypoxia in PAC by using a fluorocarbon-functionalized hollow mesoporous organosilica NPs. These NPs acted as O2 scavengers and included the IR780 sonosensitizer to improve the effectiveness of ultrasound irradiation. Co-treatment with NPs and ultrasound irradiation produced a significant increase in the concentration of oxygen and reactive oxygen species (ROS). This led to increased and maintained partial pressure of oxygen in the tumor in PANC-1 tumor-bearing mice, reversing the hypoxic state of the TME, increasing the survival rate and inhibiting tumor growth. 

Finally, some cell types present in the TME, such as macrophages, fibroblasts, and stellate cells, can promote drug resistance in the tumor due to the excessive release of cytokines and components of the ECM, such as collagen or fibronectin. Accordingly, these components have also been the target of NP-based therapeutic strategies aimed at reducing drug resistance. Tumor-associated macrophages (TAMs), which exhibit a phenotype similar to M2 polarized macrophages, limit the performance of lymphocytes and natural killers. Taking advantage of the overexpression of matrix metalloproteinases (MMPs) and esterases in the TME, Liu, et al. [178] synthesized ruthenium-based nanoplatforms functionalized with triglyceride monostearates (TGMs) for a controlled release of iBLZ-945, an inhibitor of the CSF-1/CSF-1R pathway. These nanocomplexes reversed the TAM M2 phenotype to a pro-inflammatory M1 phenotype. In fact, in CRC (CT26 cells)-bearing mice, co-treatment with these nanoplatforms and photodynamic irradiation achieved a 1.4-fold increase in CD8+ T lymphocyte infiltration. These results were explained by the reversal of the TAM phenotype to M1 resulting from the elevation of ROS levels after PDT [178]. TAMs were also used by Cao, et al. [179] to design a system based on PEG-PLGA NPs functionalized with the YI peptide to improve PTX administration. The YI peptide specifically recognizes legumarin, a surface marker of TAMs, and the ANXA1 protein present in the vasculature of the tumor. The administration of these nanoplatforms in HT-29 tumor-bearing mice resulted in high accumulation within the tumor (~3-fold) and in increased concentration of PTX (~5-fold). 

The accessibility of treatments to the tumor is hindered in PAC due to the presence of a particularly dense tumor stroma (desmoplasia), which promotes resistance. This abnormal stroma results from an excessive production of cytokines and ECM proteins, caused by the altered activity of pancreatic stellate cells (PSCs) and cancer-associated fibroblasts (CAFs). Accordingly, PSCs are another target in PAC treatment. Xie, et al. [151] synthetized multifunctional nanoplatforms, including PCX (CXCR4 antagonist), anti-miR-210 (which inactivates PSCs) and siKRASG12D (KRAS inhibitor) (PCX/(anti-miR-210+siKRASG12D). The intraperitoneal administration of these nanoplatforms in KPC8060 (mutant KRASG12D and *p53*) orthotopic tumor-bearing mice effectively decreased the expression of miR-210 (76%), Ki77+ cells (74%) and KRASG12D (74%), and reduced the expression of collagen and αSMA in PSCs. In addition, this treatment led to a greater infiltration of CD8+ T cells and a decrease in TAMs at the tumor site. By contrast, Han, et al. [180] tried to “re-educate” PSCs by using PEG-PEI-AuNPs to administer all-trans retinoic acid (ATRA) and siHSP47 (heat shock protein 47) (Au@PP/RA/siHSP47). The authors reported a 55% reduction in the expression of HSP47 in PANC-1/PSC tumor xenografts, and a lower expression of fibronectin and collagen in the ECM. In addition, co-treatment with GEM allowed to reduce the tumor weight (~60%) compared to GEM alone.

## 9. Cancer Stem Cells and Drug Resistance 

CSCs, a small subset of tumor cells with self-renewal capacity and tumorigenicity, are currently considered one of the primary causes of chemo- and radioresistance [167,181]. The main strategies developed to specifically target CSCs and eliminate resistance to treatment take advantage of the overexpression of pluripotency markers or molecules related to cell cycle alterations and cell survival.

In CRC, most of the alternatives studied involve the use of HA due to its specific binding to CD44, a marker overexpressed in this tumor. Recently, 6-mercaptopurine and thiolated HA hydrophobic based polymeric micelles selectively enhanced the effect of DOX in HCT116 colon cancer cells, showing no toxicity in normal L929 fibroblast cells [182]. Li, et al. [183] developed a multifunctional nanoplatform using RRPH (HA-PEG polymer) conjugated with the R8-RGD tandem peptide to efficiently and selectively transfect CSCs with the tumor necrosis factor (TNF)-related apoptosis-inducing ligand (TRAIL) gene associated with PF33 (fluorinated polymer). In vivo assays showed that nanoplatforms carrying the TRAIL gene were able to escape endosomes and accumulate in the nucleus of HCT116 CRC cells, significantly reducing their volume without altering other organs or modifying the blood chemistry profile. The CSC marker CD133 was the main target of the nanoplatform designed by Zahiri, et al. [184] to deliver DOX encapsulated in dendritic mesoporous silica NPs functionalized with an RNA aptamer against CD133. This nanosystem increased the toxicity of DOX in HT29 colon cancer cells as a result of greater cellular internalization. Moreover, a nanoplatform based on the ability to self-assemble of the diphtheria toxin together with the CXCR4 ligand T22 associated with 5-FU or OXA, eliminated the proliferative potential of CSC from mice tumors derived from CRC patient cells [185]. Finally, the polymeric micelles designed by Montero, et al. [186] loaded with anti-structural maintenance of chromosomes protein 2 (SMC2) antibodies and 5-FU were capable of overcoming resistance to 5-FU in HCT116 cells.

**Figure 4:**
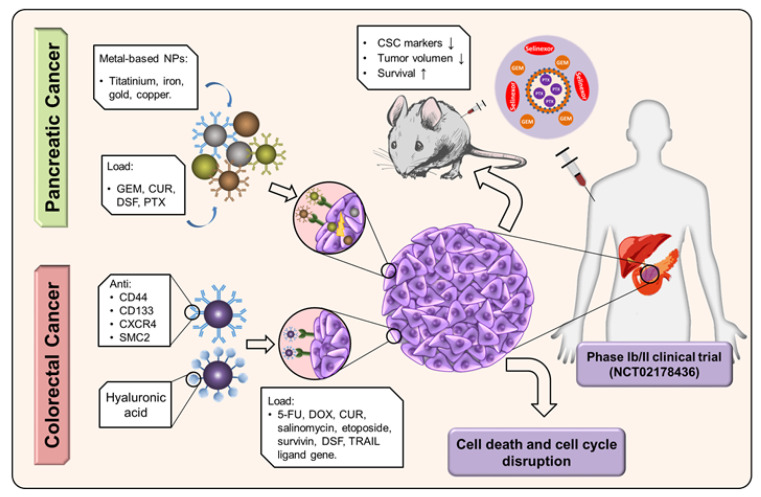
Main nanoplatforms used to overcome drug resistance in colon and pancreatic CSCs. While the CRC tends to use approaches with active targeting NPs to improve the entry of drugs, the PC opts for the use of metal-based NPs that are actively targeted or not.

In PAC, the most recent studies used metal-based nanoplatforms such as titanium, iron, gold and copper to target pancreatic CSCs. Wang, et al. [187] designed a nano-platform for PTT based on black TiO2 loaded with Gd-DOTA and targeted with anti-CD133mAb, achieving a higher and selective death of CSCs. Additionally, AuNPs [188] or curcumin-loaded superparamagnetic iron oxide NPs (SPIONs-CUR) [189] reduced resistance to GEM. AuNPs not only decreased the ability to form colonies (2D or 3D) in PAC cells, but also led to exponential decrease in the IC50 of GEM. Moreover, these NPs downregulated the expression of pancreatic CSC markers (*CD24, CD44, Epcam, CXCR4, DCLK1, nestin, CD133, c-Met, ALDH* and *Tspan8*), N-cadherin and vimentin, and upregulated E-cadherin [188]. On the other hand, SP-CUR combined with GEM, at doses at which this drug alone is not effective, selectively reduced the viability of pancreatic CSCs by 50% due to the inhibition of factors such as *Nanog*, *Sox-2*, *CD133* and *Oct-4* [189]. Also, copper-based nanoplatforms have been shown to selectively cause toxicity in CSCs by generating high levels of oxidative stress (ROS). In fact, Marengo, et al. [190] developed HA-functionalized PEG-liposomes loaded with copper complexes (Cu(DDC)2) and formed by the diethyldithiocarbamate (DDC) present in disulfiram (DSF), which reduced the DSF IC50 (2-fold) in PANC-1 CSCs. This same author used LipoCu(DDC)2–2%PEG3%HA17000 NPs to achieve a 17.5-fold reduction in the IC50 of DDC, in addition to inhibiting the ability of CSCs to form tumorspheres. Recently, Azmi, et al. [191] co-administered Selinexor and nab-PTX+GEM, improving the cytotoxic effect of the drugs and inhibiting the ability of CSCs to form tumors. In fact, a phase Ib trial using this combined therapy achieved 40% partial response and 40% disease stability rates in patients with PAC (Figure 4).

## 10. Conclusions

Gastrointestinal cancers, including CRC and PAC, frequently develop drug resistance mechanisms, preventing adequate treatment and leading to poor prognosis. Most patients with CRC, especially in metastatic stages, develop acquired drug resistance. Despite the identification of several resistance mechanisms, this phenomenon is extremely complex because it continues to develop during tumorigenesis, is dependent on the administered therapy and can be induced by several mechanisms simultaneously. Although preclinical studies combining conventional treatments with drugs such as ABC transporter inhibitors, EGFR inhibitors or autophagy inducers, have reduced drug resistance, clinical trials have not been successful. On the other hand, PAC, a refractory disease with high mortality, is also known to develop resistance, preventing drugs from exerting their antitumor effects. In this case, multiple molecular mechanisms related to resistance have been described, such as inhibition of DNA repair pathways or down-regulation of miRNA, but the development of a dense hypovascularized stroma is the essential factor underlying this phenomenon. Despite the most recent advances, clinical trials using strategies to avoid resistance have failed, although there are currently some studies in preliminary phases that try to avoid this phenomenon (Table 3). In this context, the use of novel nanoformulations seeks to provide systems that not only increase drug circulation time and accumulation in tumor tissue, but also the ability to transport therapeutic combinations with potential to overcome MDR. Of note, the development of these nanodrugs is based upon knowledge of resistance mechanisms. Accordingly, in addition to transporting drugs commonly used for colon or pancreatic cancer treatment, they allow to inhibit efflux proteins, modulate the expression of miRNA or increase the selectivity for hypoxic environments. In summary, drug resistance continues to be an obstacle for the effective application of chemotherapy. The development of new nanodrugs will undoubtedly represent an excellent therapeutic strategy to eliminate or minimize resistance in CRC and PAC.

## Figures and Tables

**Table 1:** Nanoformulations used to overcome the MDR phenotype mediated by efflux pumps in colorectal and pancreatic cancers.

Nanoformulation	Drug/Cargo	Efflux pump	CRC/PC	Mechanism to overcome MDR	Ref.
Gold nanorod coated with three layers: mSiO_2_, PHIS and TPGS	DOX	P-GP	CRC	PHIS to escape the endocytic pathway and TPGS to inhibit P-GP	[28]
Liposomes loaded with ACF	DOX	P-GP	CRC	ACF inhibits HIF-1, leading to downregulation of P-GP in hypoxic environment	[29]
Hydrogel with PEG-coated gold nanorods and TPGS-coated PTX nanocrystals	PTX	P-GP	CRC	TPGS inhibits P-GP	[30]
Vitamin E succinate-grafted-chitosan oligosaccharide with RGD and TPGS	BU	P-GP	CRC	TPGS and BU inhibits P-GP	[31]
Lignin NPs functionalized with hyaluronic acid transporting quercetin	IRI	P-GP	CRC	Quercetin inhibits P-GP	[32]
Nanovectors derived from grapefruit lipids with the LA1 aptamer and siRNA	DOX	P-GP	CRC	Downregulation of P-GP expression	[33]
Microbubbles transformable into NPs for PDT and imaging	CPT	ABCG2	CRC	Reduction of ABCG2 expression	[34]
PEG-PLGA NPs	SN38	ABCG2	CRC	Reduced mRNA expression level	[35]
Curcumin loaded in HP-β-CD	DOX	P-GP	CRC	Curcumin nanoformulation overcomes DOX resistance	[36]
Polymeric NPs with PEG and PEI	Ce6	ABCG2	PC	NPs reduce the ABCG2 efflux of Ce6	[37]
pHPMA-b-pDMAEMA NPs	ODNs	P-GP	CRC	Decreased P-GP expression by modulation of NF-κB signalling pathway	[38]
Poly (aspartic acid) with TAT peptide and PEG	DOX	P-GP	CRC	Inhibition of P-GP efflux activity by size exclusion-effect	[39]
Nanomicelles with SMA	PTX	P-GP	CRC	Enhance of drug antitumor effect with oral administration	[40]
Liposomes for PDT with benzoporphyrin derivate	IRI	ABCG2	PC	Sinergy of PDT and IRI: PDT reduces the efflux of ABCG2	[41]
PLGA NPs functionalized with Pluronic F127 and chitosan	CPT	P-GP	CRC	Pluronic F127 and chitosan downregulate MDR1 expression	[42]
Liposomes coated with hyaluronic acid	Imatinib mesylate	P-GP	CRC	Nanosystem P-GP modulation	[43]
Pegylated liposomes	ASOs and/or EPR	MDR1, MRP1, MRP2	CRC	Reduced expression of MDR1, MRP1 and MRP2	[44]
Hybrid lipid NPs with AL-HA polymer	IRI	P-GP	CRC	Disruption of ATPase activity and reduction of MDR1 gene expression	[45]
Inactive phenolato–titanium (IV) complexes	-	P-GP	CRC	Same toxicity in sensitive and resistant tumor cells	[46]
Liposomes functionalized with specific phage fusion proteins	DOX	P-GP	PC	Same drug accumulation in tumor cells in presence and absence of verapamil	[47]
Liposomes	NitDOX	P-GP, MRP1	CRC	Efflux activity reduction by nitration of P-GP and MRP1 with NO released by NitDOX	[48]
Liposomes with PEG	PTX	P-GP	CRC	Similar antitumor activity in vivo between mice bearing resistant tumor and non-resistant tumor	[49]
NPs of PEG-PLA functionalized with K237 peptide	PTX	P-GP	CRC	NPs target endothelial cells for antiangiogenic and antitumor activity in resistant tumors	[50]
Pegylated liposomes	ASOs and/or EPR	P-GP, MRP1,MRP2	CRC	Similar antitumor activity in resistant and non-resistant tumors	[51]
PLGA NPs and liposomes	GEM	NS	PC	Increase in GBC cytotoxicity in resistant tumor cell lines	[52]
Anionic liposomal NPs	DOX	P-GP	CRC	Nanosystems change the amount of P-GP lipid rafts and inhibit efflux activity (glycine 185)	[53]
PRA nanodrug coated with hyaluronic acid	-	P-GP	CRC	Generation of holes in resistant cells makes them more sensitive to DOX	[54]

Mesoporous silica (mSiO2); pH responsive polyhistidine (PHIS); acriflavine (ACF); hypoxia-inducible-factor-1α (HIF-1α); d-α-tocopherol polyethylene glycol 1000 succinate (TPGS); doxorubicin (DOX); P-glycoprotein (P-GP); colorectal cancer (CRC); pancreatic cancer (PAC); photothermal therapy (PTT); poly lactic-co-glycolic acid (PLGA); nanoparticles (NPs); verapamil (VER); polyethylene glycol (PEG); paclitaxel (PTX); arginine-glycine-aspartic acid (RGD); bufalin (BU); hydroxypropyl-β-cyclodextrin (HP-β-CD); polyethylenimine (PEI); chlorin e6 (Ce6); ATP-binding cassette subfamily G member 2 (ABCG2); NF-κB decoy oligodeoxynucleotides (ODNs); photodynamic therapy (PDT); poly[N-(2-hydroxypropyl)methacrylamide]-poly(N,N-dimethylaminoethylmethacrylate) (pHPMA-b-pDMAEMA); antisense oligonucleotides (ASOs); camptothecin (CPT); epirubicin (EPR); alendronic acid and hyaluronic acid (AL–HA); irinotecan (IRI); nitric oxide-releasing DOX (NitDOX); polyethyleneimine/all-trans retinoic acid conjugates (PRA); poly(styrene-co-maleic acid) (SMA); gemcitabine (GEM); not specified (NS).

**Table 2:** Nanoformulations including mi-RNA to overcome resistance in colorectal and pancreatic cancers.

CRC/PAC	Name	Status	Nanotransporter	Effect	Ref.
		DR			
CRC	miR-375-3p		Lipid-coated calcium carbonate with 5-FU	Inhibit TS and enhance chemosensitivity to 5-FU	[136]
	miR-200		Peptide-modified liposomes including solid lipid NPs encapsulating IRI	Increase cytotoxity of irinotecan and suppress Wnt/β-Catenin, MDR and EMT pathways	[137]
	miR-204-5p		Mesoporous silica NPs assembled with OXA and PEE/HA	Generate a synergisti effect with OXA due to an increased internalization via CD44 receptor	[138]
			PEGylated polymer NPs	Inhibit cell proliferation and promote cell apoptosis	[139]
	miR-145		PEGylated polymer NPs	Produce arrest cell cycle in GO/G1 phase, reduce tumour proliferation, migration and increase apoptosis by supressing *c-MYC*	[140]
	miR-139		Lipid polymeric NPs including Afatinib	Induce apoptosis, inhibit migration and resistance of cells via suppression of *pan-HER* tyrosine kinase	[141]
		UR			
	miR-21		Fluorescent nanodiamond	Activates *PDCD4* and *TIMP3* genes resulting in a decrease of cell invasion, migration an induction of apoptosis	[142]
	miR-155		Mesoporous silica NPs with polymerized dopamine and AS1411 aptamer	Decrease tumour growth by targeting AS1411 target (Nucleolin)	[143]
		DR			
PAC	miR-150		poly (D, L-lactide-co-glycolide)-based nanoformulation	Supress tumour growth, motility and invasion by decreasing *MUC4* and *HER2* expression	[144]
	miR-145		Magnetic NP formulation	Inhibit cell proliferation, migration and invasion by reducing *MUC13, HER2* and *pAKT* expression	[145]
	miR-216b		Palmityl-oleyl-phosphatidylcholine liposomes conjugated with cell penetrating peptide	Engage *AGO2* to promote the silencing of *KRAS* which decrease the cell proliferation and the capacity of colony formation	[146]
	miR-211		Chimeric peptide with Plectin-1 target peptides	Decrease USP99X expression and enhance DOX induced apoptosis and autophagy	[147]
	miR-9		Chimeric peptide with plectin-1 target peptides	Improve the effect of DOX through downregulating *eIF2* expression which induce apoptosis	[148]
	miR-873		Nanoliposomes	Suppress cell proliferation, migration, invasion and tumorigenesis by inhibiting the KRAS/ERK/PI3K pathways	[149]
	miR-634		Lipid NPs	Decrease the cellular proliferation by inducing apoptosis through targeting *XIAP, APIP, BIRC5*	[150]
		UP			
	miR-210		Cholesterol NPs with CXCR4 antagonist	Modulation of tumour microenvironment and inhibition of metastasis	[151]
	miR-21		PEG-PE magnetic iron oxide NPs delivered with GEM and coated of anti-CD44v6	Inhibit proliferation and metastasis by increasing *PDCD4* and *PTEN* gene expression	[152]
	miR-21-5P		Tumour penetrating NPs	Decrease the proliferation and induce apoptosis by targeting *KRAS* gene	[153]

Downregulated (DR); upregulated (UR); reference (Ref); nanoparticles (NPs); 5-fluorouracil (5-FU); oxaliplatin (OXA); gemcitabine (GEM); MDR (multidrug resistance); EMT (epithelial-mesenchymal transition); polyethyleneimine/hyaluronic acid (PEE/HA); irinotecan (IRI); Programmed Cell Death 4 protein (PDCD4); Metallopeptidase Inhibitor 3 (TIMP3); nuclear factor kappa-light-chain-enhancer of activated B cells (NF-κB); polyethylene glycol-polyethyleneimine (PEG-PEE); C-X-C Motif Chemokine Receptor 4 (CXCR4); thymidylate synthase enzyme (TS); mucin 4 (MUC4); human epidermal frowth factor receptor 2 (HER2); mucin 3 (MUC13); AKT Serine/Threonine Kinase 1 (pAKT1); argonaute-2 (AGO2); eukaryotic initiation factor 2 (EIF2); x-linked inhibitor of apoptosis (XIAP); APAF1 interacting protein (APIP); Baculoviral IAP repeat containing 5 (BIRC5); phosphatase and tensin homolog (PTEN); cellular transforming proto-oncogene (KRAS).

**Table 3:** Clinical trials using NPs designed against CRC and PAC to overcome multidrug resistance.

Identifier	Drug/Cargo	Clinical Phase	PAC/CRC
NCT02178436	Selinor + GEM + nab-paclitaxel	Phase Ib	PAC
NCT02010567	CRLX101-capecitabine + radiotherapy	Phase I/II	CRC
NCT00081549	Aroplatin (liposomal NDDP) + GEM	Phase I/II	PAC
NCT00043199	Aroplatin (liposomal NDDP)	Phase II	CRC
NCT00081536	Aroplatin + capecitabine	Phase I/II	CRC
NCT03883919	IRI+5-FU/LV + paricalcitol	Pilot Study	PAC
NCT03337087	Liposomes transporting IRI, 5-FU, LV and rucaparib	Phase I/II	PAC and CRC

Gemcitabine (GEM); bis-neodecanoate diaminocyclohexane platinum (NDDP); irinotecan (IRI); 5-fluorouracil (5-FU); leucovorin (LV).
